# Draft Genome Sequence of *Vibrio coralliilyticus* Strain OCN008, Isolated from Kāne‘ohe Bay, Hawai‘i

**DOI:** 10.1128/genomeA.00786-13

**Published:** 2013-10-03

**Authors:** Blake Ushijima, Patrick Videau, Greta S. Aeby, Sean M. Callahan

**Affiliations:** Department of Microbiology, University of Hawaiˈi, Honolulu, Hawaiˈi, USAa; Hawaiˈi Institute of Marine Biology, Kāneˈohe, Hawaiˈi, USAb

## Abstract

*Vibrio coralliilyticus* is a Gram-negative bacterium found in seawater and is associated with diseased marine organisms. Strains of *V. coralliilyticus* have been shown to infect coral from multiple genera. We report the draft genome sequence of *V. coralliilyticus* strain OCN008, the third *V. coralliilyticus* genome to be sequenced.

## GENOME ANNOUNCEMENT

*Vibrio coralliilyticus* is a marine gammaproteobacterium that has been implicated as a pathogen in diseases that affect marine organisms (1–4). It has a broad host range that includes the corals *Pocillopora damicornis* (1), *Pachyseris speciosa*, *Montipora aequituberculata*, and *Acropora cytherea* (4). Infection results in tissue loss and/or bleaching (loss of symbiotic algae from the coral), depending on the temperature and coral host (5). *V. coralliilyticus* also causes disease in the mussel *Perna canaliculus* (3) and the rainbow trout *Oncorhynchus mykiss* (2). The geographic range of *V. coralliilyticus* is also broad; infections attributed to *V. coralliilyticus* have been reported in the Indian Ocean (6), Red Sea (5), Caribbean (7), north Atlantic Ocean (2), and south Pacific Ocean (4). Despite the global distribution and infectious capacity of this bacterium, only two *V. coralliilyticus* genomes are currently available, those for strains BAA-450 (accession no. ACZN00000000) and P1 (accession no. AEQS00000000).

*V. coralliilyticus* strain OCN008 was isolated from the coral *Porites compressa* in Kāne‘ohe Bay, Hawai‘i. A fragment of *P. compressa* taken from a fringing reef of Moku o Lo‘e was crushed with mortar and pestle in sterile seawater and plated for single colonies on solid glycerol artificial seawater medium, prepared as previously reported (8). This particular strain was of interest based on a zone of apparent growth inhibition that surrounded the colony. Genomic DNA was isolated from strain OCN008 using a phenol-chloroform extraction method and sequenced using the Roche 454 GS FLX Titanium system and Ion personal genome machine sequencer technology at the Advanced Studies of Genomics, Proteomics, and Bioinformatics Core Facility (Honolulu, HI; http://asgpb.mhpcc.hawaii.edu). The high-throughput sequencing reads yielded 441 Mb of sequence (approximately 80-fold coverage), and the 2,672,587 reads were assembled using the Newbler software (version 2.8) into 210 contigs with an average contig size of 26.4 kb. Annotation was conducted using the NCBI Prokaryotic Genome Automatic Annotation Pipeline. General analysis was conducted using the Rapid Annotations using Subsystems Technology (RAST) server (9).

The draft genome consists of 5,534,904 bp, has a 45.7% G+C content, and contains 5,436 genes. Of the total genes, 2,517 nonhypothetical and 170 hypothetical genes (48% of total) were categorized into 516 metabolic subsystems. Of interest are 117 genes that are predicted to be involved in virulence, disease, and defense. A total of 45 tRNA and 4 rRNA coding sequences were annotated.

### Nucleotide sequence accession numbers.

This whole-genome shotgun project has been deposited at DDBJ/EMBL/GenBank under the accession no. AVOO00000000. The version described in this paper is version AVOO01000000.
